# Challenges Facing Asian Sex Workers in Western Australia: Implications for Health Promotion and Support Services

**DOI:** 10.3389/fpubh.2018.00171

**Published:** 2018-06-13

**Authors:** Linda A. Selvey, Roanna C. Lobo, Kahlia L. McCausland, Basil Donovan, Julie Bates, Jonathan Hallett

**Affiliations:** ^1^Division of Disease Prevention and Control, School of Public Health, Faculty of Medicine, The University of Queensland, Brisbane, QLD, Australia; ^2^Collaboration for Evidence, Research and Impact in Public Health, School of Public Health, Curtin University, Bentley, WA, Australia; ^3^The Kirby Institute, University of New South Wales, Sydney, NSW, Australia; ^4^Sydney Sexual Health Centre, Sydney Hospital, Sydney, NSW, Australia; ^5^Urban Realists Planning and Health Consultants, Sydney, NSW, Australia

**Keywords:** sex worker, prostitution, migrant, stigma, discrimination, legislation, sex industry, health and safety

## Abstract

**Introduction:** Asian sex workers are a significant part of the Australian sex industry. Criminal laws, racism, isolation, poor English language skills and stigma and discrimination combine to increase the vulnerability of Asian sex workers in Australia. To inform service delivery and potential legislative reform, we undertook a study of sex worker health and safety in Western Australia with a focus on Asian sex workers.

**Methods:** This was a mixed methods study in which peer researchers played an essential role. We undertook a survey (available online and in paper form and translated into three languages other than English), semi-structured interviews with sex workers, and interviews with key advisors.

**Results:** In our study, Asian sex workers were older, had lower levels of education, more likely to have sex work as their main source of income, work longer hours and work exclusively in a shop-front massage parlor compared to their non-Asian counterparts. The vast majority of Asian sex workers in our study said they had poor English language skills and the greatest proportion spoke Chinese languages. Sex work had a positive impact on the well-being of many respondents, and their level of psychological distress was similar to the general Australian population. Stress and “bad clients” were common negative impacts of sex work. Asian study participants were less likely than their non-Asian counterparts to smoke, undertake risky drinking or use illicit drugs. A similar proportion of Asian sex workers reported being assaulted compared to their non-Asian counterparts.

**Discussion/Conclusion:** The major challenges facing Asian sex workers in WA seem to be stigma and discrimination, stress, social isolation, and confusion about their legal standing leading to a fear of authorities, particularly the police. Our findings support the need for enhanced targeted peer-based health promotion outreach services for Asian sex workers, increased Asian language services in sexual health clinics and decriminalization of sex work.

## Introduction

Asian sex workers form a significant part of the Australian sex industry. The proportion of sex workers in Sydney accessing sexual health clinics who were from Asia increased from 20% to over 50% from 1992 to 2009 (1). This was also reflected in other jurisdictions including Western Australia (WA) (2). Currently the majority of Asian sex workers are from China, Thailand and Korea (1–3).

Earlier studies found relatively low rates of condom use with clients and high rates of sexually transmissible infections (STI) among Asian sex workers (4–6). In Australia at least, this improved as a result of health promotion, with recent studies suggesting that reported condom use and STI rates were comparable between Asian and non-Asian sex workers (3, 5, 7).

The intersection of criminal laws, racism, isolation, poor English language skills and stigma and discrimination associated with sex work potentially increases the vulnerability of Asian sex workers in Australia. These factors may also result in a reluctance to access services or associate with peers from the same country (8). Potential vulnerabilities could include increased risk of assault, social isolation, and poorer sexual health outcomes. It is therefore important to understand the needs of Asian sex workers so that, where required, accessible and acceptable support and services can be provided.

A study of the influence of sex work legislation on the health and safety of sex workers was undertaken in WA, New South Wales and Victoria in 2007; three states in Australia that each have different legislative frameworks in relation to sex work (9). These frameworks are criminalization, decriminalization, and licensing respectively (1, 9). The *Prostitution Act 2000* and the *Criminal Code* govern prostitution law in WA. The *Prostitution Act 2000* states that sex work is not an offense. However, it is illegal under the Prostitution Act to live on the earnings of another's sex work. Street-based sex work is illegal in WA under the *Prostitution Act 2000* and the *Criminal Code* explicitly disallows keeping or managing a brothel (10). Regardless, there are a number of highly visible brothels in Perth and at least one regional center, and criminal sanctions tend to focus predominantly on street-based sex work (2, 9). In this study we built on the earlier study in order to describe changes in the WA sex industry in the later decade. We also extended the study to involve sex workers across the full range of sex industry businesses that exist in WA. These include brothel based, escort, private, and street based sex work including outside of the Perth metropolitan area, and male and transgender workers. Given the increase in numbers of Asian sex workers over the previous 10 years, we aimed to recruit to our study a significant number of Asian sex workers working in a range of workplace settings. This paper describes our findings in relation to Asian sex workers.

## Methods

This was a cross-sectional mixed methods study that included visits to brothels and other sexual services premises, a survey of sex workers, interviews with key advisors from within and outside the sex industry, and in-depth interviews with sex workers.

### Peer researchers

Eight sex workers and one brothel receptionist were recruited as peer researchers through our networks including formal and informal contacts and were trained by an investigator (JB). The peer researchers included one white Australian male, three white Australian females, and five female Asian peer researchers (two Chinese, two Thai and one Korean).

### Visits to brothels and other sexual services premises

Peer researchers undertook all visits to sexual services premises. The process for selecting and visiting premises has been described elsewhere (2). Regional visits to Kalgoorlie, Rockingham, Bunbury and Mandurah were undertaken by a peer investigator (JB) during which attempts were made to identify and visit sexual services premises. Because we anticipated challenges with identifying and gaining entry to sexual services premises with predominantly Asian sex workers, we attempted to visit all such premises including a number of shop-front massage parlors and other sexual services premises that were identified by peer researchers.

### Key advisor interviews

Key advisors included sex workers, representatives from sex worker organizations, brothel and sexual services premises owners or managers, and representatives from sexual health clinics, the WA police and WA local governments. Key advisors were identified through researchers' networks. A random selection of local governments were invited to participate in an interview. The interviews were semi-structured, and when consent was given, were recorded and transcribed.

### Sex worker survey and survey recruitment

The sex worker survey instrument was based on a survey that was used previously to survey sex workers in brothels in Perth, Sydney and Melbourne (9). Additional questions were included in the survey following consultation with key stakeholders including peer-based sex worker organizations. The survey included the Kessler K10 scale (11, 12), and a validated question relating to binge drinking (13). The survey was self-administered either online or on paper and included a range of questions including about interactions with police and experiences of violence and stigma (2). The survey was translated into Korean, Thai and Chinese by NAATI (National Accreditation Authority for Translators and Interpreters) accredited personnel and checked by peers for appropriate use of language and context. Participants received AUD30 for a completed survey.

Survey respondents were recruited by several means including via social media; advertisements in print media; notifications to e-lists and sex worker organizations; visits to sexual services premises; and peer networks. The majority of Asian sex worker survey respondents were recruited by peer researchers, either via their networks or their visits to sexual services premises.

Free text responses to the survey were given in the above languages and were translated into English and manually entered using SPSS v24 (IBM Analytics, New York, USA).

### In-depth interviews with sex workers

Participants for in-depth interviews were recruited by peer researchers via their networks. Semi-structured in-depth interviews were conducted after the survey data were analyzed using an interview guide based on themes of interest arising from the survey results. Asian interview participants were recruited through word of mouth by peers and peer-based organizations.

The interviews were conducted by four investigators (LS, KM, JH, and RL) either in person or over the phone with interpretation assistance by a peer where necessary. Of the 17 interviewees, five were Asian (four female, one male). Interviews lasted between 30 and 90 min and all but two were audio-recorded and transcribed verbatim. Participants received AUD50 in cash for their participation. Only data from in-depth interviews with Asian participants are described here.

### Data analysis

Quantitative: Analysis of the survey responses involved frequency analyses and Mantel Haenszel Chi squared or where appropriate Fischer's Exact Test were used to estimate p values for comparisons between groups. The responses to the Kessler K10 scale were scored as described previously (11, 14). Scores can range from 10 to 50. Scores of less than 20 were considered to be indicative of low; 20 to 24 of mild; 25 to 29 of moderate; and scores of 30 and over of severe psychological distress (11, 14). Compared to non-Asian respondents, a smaller proportion of Asian respondents were male. To avoid potential confounding, when comparisons between Asian and non-Asian respondents were made, only data from female respondents were included in the analysis where there were differences between male and female respondents in the overall study sample. Data analysis was undertaken using SPSS v24 (IBM Analytics, New York, USA).

Qualitative: Survey free text responses and interviews with Asian sex workers were included in the analysis. Free text responses to questions in the survey were translated into English and included in the thematic analysis. Thematic analysis involved reading the interview transcripts and free text responses several times and noting down points of interest. Descriptive codes were assigned to the points of interest and then were grouped into categories to develop the overarching themes (2, 15). Data management and coding was conducted using NVivo v11 (QSR International).

### Ethics

This study was carried out in accordance with the recommendations of the National Statement on Ethical Conduct in Human Research, developed jointly by the National Health and Medical Research Council, Australian Research Council and the Australian Vice-Chancellors' Committee (2007). The protocol was approved by the Curtin University Human Research Ethics Committee, approval number HRE2016-0078. All subjects gave informed consent in accordance with the Declaration of Helsinki.

## Results

### Survey recruitment

There were 354 survey respondents, of whom 94 (27%) reported that their country of birth was in Asia. Of those 94 respondents, 85 (90%) responded to surveys in languages other than English; 54 in Chinese, 22 in Thai and nine in Korean. Only 14 Thai surveys and three Chinese surveys were completed online (20%). This is in contrast to the English surveys, of which 73% (196) were completed online (*p* < 0.001). Recruitment in regional areas was challenging. While the identification of, and visits to premises was undertaken by a peer investigator, she was denied entry to a number of unmarked “private houses” (most proprietors would not even open the door), and some massage shop proprietors denied that sexual services were being provided on their premises. This may reflect language constraints and a fear of being closed down or of immigration authorities.

“*I don't like to meet anyone other than the clients. No police officers. No immigration officials.”* (Asian sex worker survey respondent number 19, female).

### Demographics of survey respondents

The majority of Asian survey respondents (92%) identified as female and 55% spoke Chinese at home (Table 1). A higher proportion of Asian respondents (31%) reported working exclusively in massage parlors compared to non-Asian respondents (6%, *p* < 0.001). The majority of Asian sex workers commenced sex work when they came to Australia. Of the 90 Asian sex workers who responded to the question, 14 (16%) had previously engaged in sex work overseas. A number of respondents, and two in-depth interview participants described coming to Australia to either study, have a working holiday, or for the experience, and ended up doing sex work because of difficulty in finding other work that pays enough, particularly because of poor English skills.

“*You can have money, negative: I don't know English, I don't know what work I can do.”* (Asian sex worker survey respondent number 61, female).

“*I came to Australia, my English is not good, if I look for other work the income is very low and I can't support my tuition and living cost. Therefore, I do massage work and I can make some pocket money.”* (Asian sex worker survey respondent number 64, female).

**Table 1:** Demographics of Asian sex worker survey respondents.

**Characteristic**	**Asian respondents n (%)**	**Non-asian respondents n (%)**	***p*-value^a^**
Gender			
Male	6 (6.6)	46 (17.9)	
Female	84 (92.3)	199 (77.4)	
Genderqueer	1 (1.1)	10 (3.9)	0.008
Sex assigned at birth			
Male	10 (10.6)	58 (22.3)	
Female	84 (89.3)	202 (77.7)	0.014
Age			
18–25 years	9 (9.6)	78 (30.6)	
26–35 years	48 (51.0)	103 (40.4)	
36–50 years	32 (34.0)	61 (23.9)	
51+ years	5 (5.3)	13 (5.1)	0.001
Language spoken at home			
Chinese	52 (55.4)	–	
Thai	22 (23.4)	–	
Other Asian	20 (21.3)	–	
English language skills fair or poor	88 (93.6)	80 (31.1)	<0.001
Highest level of education			
Primary school or			
equivalent	10 (10.6)	4 (1.6)	
High school or equivalent	45 (47.9)	102 (40.6)	
Post-high school	39 (41.5)	145 (57.8)	0.001
Has Medicare entitlements	40 (43.5)	168 (65.6)	0.001
Duration of sex work (australia or overseas)			
Less than 1 year	10 (11.1)	40 (16.1)	
1 to 2 years	38 (42.2)	69 (27.7)	
More than 2 years	42 (46.7)	140 (56.2)	0.016
Does at least some private work	37 (39.4)	159 (61.2)	<0.001
Works in a brothel at least some of the time	20 (21.3)	63 (24.2)	0.562
Works in a massage shop at least some of the time	41 (43.6)	78 (30.0)	0.017
Works exclusively in massage shops	29 (30.9)	15 (5.8)	<0.001

a *p-value for the comparison between Asian and non-Asian sex workers*.

Of 92 Asian respondents, 40 (43.5%) reported having Medicare entitlements and seven (8%) were unsure about their health insurance status. Of 93 Asian respondents, 73 (78.5%) reported that sex work was their main source of income, which was higher than non-Asian respondents (62%, *p* = 0.003). When male respondents were excluded from the analysis, this difference remained with 66 of 83 Asian female respondents (80%) and 126 of 197 non-Asian female respondents (64%) reporting that sex work was their main source of income (*p* = 0.010).

Compared to their non-Asian female counterparts, a higher proportion of Asian female respondents reported seeing more than 20 clients and working more than 40 h per week in sex work (Table 2).

**Table 2:** Number of hours worked and clients seen in an average week by female respondents.

	**Asian sex workers *n* (%)**	**Non-asian sex workers *n* (%)**	***p*-value**
**Number of clients**	*N* = 56	*N* = 201	
Less than 10	5 (8.9)	67 (33.3)	
10–20	30 (53.6)	95 (47.3)	
21–30	11 (19.6)	24 (11.9)	
Greater than 30	10 (17.9)	15 (7.5)	<0.001
**Number of hours worked (sex work)**	*N* = 57	*N* = 232	
Less than 20	12 (21.1)	94 (40.5)	
20–40	21 (36.8)	104 (44.8)	
41–50	11 (19.3)	20 (8.6)	
Greater than 50	13 (22.8)	14 (6.0)	<0.001

### Sex worker well-being

Eighty four Asian respondents (89%) completed the Kessler K10 questionnaire items in the survey. Of these, 51 (61%) had a score consistent with low psychological distress, and a further 13 (16%) with mild psychological distress. This is in contrast to the non-Asian respondents; of the 217 respondents, 89 (41%) had a score consistent with low psychological distress and 38 (18%) with mild psychological distress (*p* = 0.015).

Having a good income, including being able to support their families and having work flexibility was often cited by respondents as being ways that sex work enhanced their well-being.

“*Positive side is that I feel happy that I help my family to reduce burden.”* (Asian sex worker survey respondent number 34, female).

“*I can send money to Thailand, my family can have a good life.”* (Asian sex worker survey respondent number 46, female).

“*For me sex work is suitable because I need money and flexible working hour. And now I am also a full time student. I can make money working and studying. Time more flexible.”* (ID16 in-depth interview).

Some Asian respondents described long working hours, job stress and demanding clients as having a negative impact on their well-being.

“*I think it hinders my wellbeing sometimes. E.g. Lack of exercise sometimes or overwork sometimes. I don't see the sunlight much.”* (Asian sex worker survey respondent number 19, female).

“*As I deal with so many people, it is stressful and I think that my health will be harmed.”* (Asian sex worker survey respondent number 37, female).

“*The clients are mostly demanding and aggressive. I have to do my job even though I sometimes do not feel like it.”* (Asian sex worker survey respondent number 54, female).

Twenty nine percent of Asian respondents reported currently smoking cigarettes, and 23% reported harmful alcohol use (Table 3). These proportions were lower than non-Asian respondents (Table 3). With the exception of alcohol consumption, these differences were also significant when only female respondents were included in the analysis (data not shown).

**Table 3:** Reported current drug and alcohol use in the previous year.

	**Asian sex workers *n* (%)**	**Non-asian sex workers *n* (%)**	***p-*value**
Cigarettes	27 (28.7)	128 (49.4)	0.001
At least six standard drinks on one occasion weekly or more	20 (22.5)	90 (38.0)	0.008
Marijuana	2 (2.1)	38 (14.7)	0.001
Methamphetamines	1 (1.1)	29 (11.2)	0.004
Injected a drug in the past 12 months	4 (4.5)	35 (14.5)	0.020

### Condom use and sexual health

Forty of 90 (44%) Asian respondents reported that they learned about safe sex and sex work skills nowhere or from clients (that is, they did not learn about safe sex and sex work skills from sources such as the internet, other workers, information booklets, health services or peer educators). This was a higher proportion than non-Asian respondents (23%, *p* < 0.001). However, there was no difference between female Asian and non-Asian respondents in reported condom use with clients (Table 4).

**Table 4:** Condom use with clients for female sex workers who provided this service to clients.

	**Asian sex workers *n* (%)**	**Non-asian sex workers *n* (%)**	***p-*value**
**Oral sex**	*N* = 69	*N* = 173	
All clients use condoms	24 (34.8)	61 (35.3)	
Most clients use			
condoms	18 (26.1)	34 (19.7)	0.399
**Vaginal sex**	*N* = 68	*N* = 173	
All clients use condoms	50 (73.5)	115 (66.5)	
Most clients use			
condoms	15 (22.1)	51 (29.5)	0.898

Of 60 Asian respondents who did not work exclusively in massage parlors, 34 (57%) reported having had a sexual health check in the past three months. This was a lower proportion than non-Asian respondents, of whom 74% (168/228) reported having had a sexual health check in the past 3 months (*p* = 0.010). This difference remained if only female sex workers were included in the analysis (55 vs. 73%, *p* = 0.012).

Having the ability to access appropriate health and other services appeared to be a priority for some respondents, but this was hampered by a lack of resources and support in their own languages, the requirement for having a Medicare card for some services, stigma and long waiting times. Mental health support was another service that some respondents mentioned as important.

“*I feel that my physical condition is not good due to my work but I do not know what to do and where to go. In fact I am rather worried. I wish there were a special clinic just for us.”* (Asian sex worker survey respondent, 7, female).

“*I would like to receive regular check-ups in an easier way without needing to use a card.”* (Asian sex worker survey respondent, 39, female).

### Isolation, stigma, racism

A number of Asian sex workers reported avoiding disclosure of the nature of their work to family, friends and professionals out of fear of experiencing stigma and discrimination, and in particular having their families and people in their home towns or villages become aware of their profession. While this is not uncommon with other sex workers in Australia, isolation appeared to be more pronounced for Asian sex workers. In their responses, a fear of being “looked down upon” was a common phrase that Asian survey respondents used to describe their concerns about what may happen if other people found out about their work.

“*I'm afraid of being found out by families and being looked down upon.”* (Asian sex worker survey respondent 67, female).

“*People will discriminate and be embarrassed to have me in the community.”* (Asian sex worker survey respondent 11, male).

“*I'm scared of making new friends. I am afraid of being asked about my work. I am afraid of meeting someone who I know.”* (Asian sex worker survey respondent 65, female).

“*Doing this type of work I become isolated because I don't want to reveal myself to the society. In Thai culture, the majority of the people, who know that anyone is a sex worker, will take offence and will not let that person join any activity in the society.”* (Asian sex worker survey respondent 47, female)

“*This is my country's culture. If Australia people you do this job you can tell your family and your friends or not. Because this is my country's culture, my culture is very very important. Hong Kong is a little bit OK but China no way, no way.”* (ID16, in depth interview).

As well as resulting in social isolation, the fear of experiencing stigma and discrimination can impact on the ability to get information and support in relation to work-related issues, as described below.

“*If there is an incident, a condom breaking while having sex with a customer, where should I go for consultation? I like to keep it secret. Where can I get any information and news?”* (Asian sex worker survey respondent 41, female).

Having a lack of a support network could be an indication of isolation. Of 92 Asian respondents, 48 (52%) reported having a support network, compared to 67% of 254 non-Asian respondents (*p* = 0.006). Finding a support network outside of work is challenging because of fear of disclosure and working long hours. Some Asian sex workers described finding a support network within the work setting as also challenging, in part due to a sense of competition with other workers and a lack of trust in each other.

An interview participant who worked in a small “private house” described not having someone to talk to if she had a problem apart from her workmates, but that her workmates are not friends, they “just talk.” She described some other workers as being “sometimes selfish” and “stealing her turn” (for a customer), although some others do look out for her. (ID09, in depth interview, not recorded).

“*Yeah I usually talk with my friend. To be honest, to be a sex worker is difficult to find a friend. It [is] really difficult to make [a] friend with other girls working in the shop especially when they come from China or same culture… And now the sex industry to make money is much more difficult… they want to get more jobs. So I never tell the girls what kind of service I provide.”* (ID15 in depth interview).

A number of respondents who work privately described experiencing discrimination by landlords and having to pay considerably more rent for premises because of their work. This may not be different from the experiences of non-Asian sex workers, but it may be exacerbated by language difficulties and/or racism.

“*There should be a law that does not discriminate in rental areas when it comes to sex workers. Most owners want to charge three or four times the market rent when they know that the premises will be used for sex work. If you refuse to pay they threaten to go to the police. However for any other business, the rules are different.”* (Asian sex worker survey respondent 59, female).

### Assault and confidence in the police

Of the 59 Asian sex workers who responded to this question and who did not work exclusively in massage parlors, 21 (36%) reported having been threatened; 14 (24%) reported having been assaulted; and 28 (48%) reported having been pressured to do something they didn't want to do by a client in the past 12 months. This is similar to the responses of the non-Asian respondents (*p* = 0.314, 0.997 and 0.184 respectively).

The majority of Asian respondents (59/86, 69%) reported feeling uncomfortable or very uncomfortable in reporting to the police assaults, threats and thefts in the course of their work. This was significantly higher than the 42% of non-Asian respondents (102/243) (*p* < 0.001) reporting feeling uncomfortable or very uncomfortable in going to the police. For some respondents the level of discomfort in going to the police related to a fear of discrimination or of not being taken seriously; losing their visa, or experiencing racism. Having the perception that sex work is illegal was also a barrier to reporting crimes to the police. In addition, a number of Asian survey respondents and an Asian study advisor expressed a desire for more protection for sex workers.

“*Because of our special job, when we were attacked we normally disguise a big incident as a minor one and dismiss a minor incident. Many sex workers when being attacked would not be willing to report to the police.”* (Asian sex worker survey respondent 24, female).

“*Because even if I report to the police, the police arrives. If I cannot provide enough evidence, the criminals still cannot be brought to justice.”* (Asian sex worker survey respondent 24, female).

“*If there is any incident, get threatened by a customer, want a police to come and help in time.”* (Asian sex worker survey respondent 41, female).

In contrast, one Asian respondent reported that they had contacted the police after experiencing a problem with a client and that the police were helpful.

When describing clients, most Asian respondents described having good or considerate clients as well as not so good clients, who could be rough and difficult. One Chinese sex worker described how she managed to prevent clients from getting angry as she felt she was unlikely to get support from her boss.

“*I work Chinese shop. I don't want to make customer angry. If customer angry he will tell my boss. My boss won't want me to work in this shop again… Ah I'm clever. I do not want to make annoying to anyone*.” (ID15, in-depth interview).

One interview participant described racism that she experienced from some clients as well as outside of the work setting, such as in her local shopping center. She described some clients being verbally abusive and “rough” rather than threatening or assaulting her. While opportunities to screen clients over the phone are limited for some Asian sex workers due to English language difficulties, information about “bad clients” is shared with other sex workers so that those clients can be avoided.

## Discussion

Compared to non-Asian survey respondents, the Asian sex workers in this study were older, less well-educated, more likely to have sex work as their main source of income, work longer hours and were more likely to work exclusively in a shop-front massage parlor. Other Australian studies have also found that Asian respondents tended to be older and have lower education levels than their non-Asian counterparts (3). The vast majority of Asian sex workers in our study said they had poor English language skills and the greatest proportion spoke Chinese languages. Sex work had a positive impact on the well-being of many respondents. Stress and “bad clients” were common negative impacts of sex work. In our study, the level of psychological distress among Asian sex workers was similar to the general Australian population (14), and was lower than their non-Asian counterparts. This may reflect lower levels of alcohol and drug use (9). Asian study participants were less likely than their non-Asian counterparts to smoke, undertake risky drinking or use illicit drugs.

A similar proportion of Asian sex workers reported being assaulted compared to their non-Asian counterparts. However, a high proportion (69%) of survey respondents reported feeling uncomfortable with seeking assistance from the police in the event of an assault or other crime against them. Uncertainties about the legislation around sex work, concerns about experiencing racism from police or being reported to immigration were reported barriers to seeking police assistance. These concerns may also explain the unwillingness of proprietors of massage shops to admit to providing sexual services and the difficulty in gaining access to Asian sexual services premises (unless as a client) without having previously built a relationship with them. These are barriers to providing health promotion and support services, and demonstrate the importance of having Asian peer-based support services located in regional areas as well as in Perth, in order to have the time to build trust.

Social isolation was a significant issue faced by a number of Asian sex workers. Many migrants face some level of social isolation as a result of disruption to their social networks. The social isolation described by our study participants is exacerbated by significant stigma and discrimination against sex workers in Australia and even more so in their countries of origin, and a fear of being “found out” by family and friends at home. This is particularly the case for Chinese sex workers. A sense of competition with other sex workers in the workplace also seemed to exacerbate the isolation for some. This sense of competition may be enhanced by the downturn in the sex industry that has accompanied a downturn in the WA economy (2).

Potential consequences of this social isolation include stress and an unwillingness to access, or lack of knowledge about access to support services such as peer-based sex worker organizations. We found that Asian survey respondents were more likely to report not accessing safe sex information from anywhere other than clients compared to non-Asian respondents, which may reflect this isolation as well as a relative lack of such information available in Asian languages. Compared to non-Asian respondents, a lower proportion of Asian respondents reported having a sexual health check in the past three months even when respondents working exclusively in massage parlors were excluded. Again, this may reflect relative isolation as well as reduced access to health services due to not being eligible for Medicare.

The Asian survey respondents reported similar rates of condom use to non-Asian respondents. In contrast, a study of female sex workers at the Sydney Sexual Health Centre found that sex workers speaking Mandarin or Cantonese were more likely, and Thai speakers less likely to report inconsistent condom use during oral sex with clients (16). We did not have sufficient sample size to investigate condom use by language spoken or service location.

Other studies have investigated the health and welfare of Asian sex workers in Australia (3, 5, 8, 17), predominantly in brothel and massage parlor settings. Our study was unique in that almost 40% (37) of our survey respondents did at least some private sex work. This reflects our survey recruitment as all but one (5) of the other studies targeted sexual services premises for survey recruitment. We also utilized peer researchers for study recruitment and participants could complete their surveys either online or on paper; thus also increasing the chances of obtaining a more diverse study sample. Our study sample is also more reflective of the overall sex industry, with increasing proportions of sex workers working privately (2). In spite of these differences in the study sample, our findings were similar to those of other studies. As well as participant demographics, the experiences of stigma and discrimination and social isolation were also reported in other studies (3, 8), as was our finding of relatively low drug and alcohol use compared to non-Asian sex workers (1, 3, 5).

Our results point to the need for enhanced targeted peer-based health promotion, education and support services for Asian sex workers in Perth and regional locations. This should include interventions such as training of police recruits to enhance the relationship between Asian sex workers and the police in order to increase the willingness to report crimes against them. Enhanced Asian language services in sexual health clinics (access to Medicare is not required to access sexual health clinics) would increase the accessibility of these services. Decriminalization of sex work in WA and elsewhere in Australia would also contribute to increasing the visibility of some Asian sexual services premises to enable outreach health promotion, education and support services and enable open promotion of safe sex messages in sexual services premises. Decriminalization may also increase the willingness of Asian sex workers to report to the police crimes committed against them in the workplace.

### Limitations

The study sample was not randomly selected and the selection process means that it is likely to be biased in favor of Asian sex workers who are more connected with existing peer support services. The fact that the majority of Asian-language surveys were completed on paper whereas the majority of English-language surveys were completed online may reflect a difference in recruiting between Asian and non-Asian respondents, with Asian respondents being more likely to be recruited via direct contact with a peer researcher than non-Asian respondents. An alternative explanation would be that, compared to non-Asian respondents, Asian survey respondents may have less access to or be mistrustful of providing online information. Regardless, it does reflect the importance of peer-based health promotion and support services for Asian sex workers.

We did not collect biological specimens from our study participants in order to test for STIs. However, recent Australian data in relation to STIs among sex workers is available, and shows a rising incidence of STIs among sex workers that is consistent with the rise in incidence among the general population (7, 18).

## Conclusions

The major challenges facing Asian sex workers in WA seem to be stigma and discrimination, stress, social isolation, and confusion about their legal standing leading to a fear of authorities, particularly the police. Our findings support the need for enhanced targeted peer-based health promotion outreach services for Asian sex workers, increased Asian language services in sexual health clinics and decriminalization of sex work.

## Author contributions

LS led the overall study, analyzed the data and wrote the manuscript. JH, KM, JB, and RL contributed to data collection and interpretation. BD contributed to data interpretation. All authors contributed to study design and read and approved the final manuscript.

### Conflict of interest statement

The authors declare that the research was conducted in the absence of any commercial or financial relationships that could be construed as a potential conflict of interest.
